# Heavy metal in radiology: how to reliably differentiate between lodged copper and lead bullets using CT numbers

**DOI:** 10.1186/s41747-020-00168-z

**Published:** 2020-07-06

**Authors:** Dominic Gascho, Niklaus Zoelch, Henning Richter, Alexander Buehlmann, Philipp Wyss, Michael J. Thali, Sarah Schaerli

**Affiliations:** 1 Department of Forensic Medicine and Imaging, Institute of Forensic Medicine, University of Zurich, Winterthurerstrasse 190/52, CH-8057 Zurich, Switzerland; 2 Department of Psychiatry, Psychotherapy and Psychosomatics, Hospital of Psychiatry, University of Zurich, Zurich, Switzerland; 3 Diagnostic Imaging Research Unit (DIRU), Clinic for Diagnostic Imaging, Vetsuisse Faculty, University of Zurich, Zurich, Switzerland; 4 Zurich Forensic Science Institute, Zurich Canton Police and Zurich City Police, Zurich, Switzerland; 5 Institute of Forensic Medicine, Health Department Basel, University of Basel, Basel, Switzerland

**Keywords:** Copper, Forensic ballistics, Lead, Tomography (x-ray computed), Wounds (gunshot)

## Abstract

**Background:**

The *in situ* classification of bullets is of interest in forensic investigations when the bullet cannot be removed. Although computed tomography (CT) is usually performed on shooting victims, visual assessment, or caliber measurements using CT can be challenging or infeasible if the bullets are deformed or fragmented. Independent from the bullet’s intactness, x-ray attenuation values (CT numbers) may provide information regarding the material of the bullet.

**Methods:**

Ethical approval was not required (animal cadavers) or waived by the ethics committee (decedents). Copper and lead bullets were fired into animal cadavers, which then underwent CT scanning at four energy levels (80, 100, 120, and 140 kVp). CT numbers were measured within regions of interest (ROIs). In addition to comparing CT numbers, the dual-energy index (DEI), representing the ratio between the CT numbers of two energy levels, was calculated. The most appropriate method was applied for decedents with fatal gunshot wounds.

**Results:**

CT numbers demonstrated no significant difference between copper and lead bullets, and false classifications can easily occur. DEI calculations revealed significant differences between the two groups of bullets. The 120/140 DEIs calculated from the maximum CT numbers obtained from ROIs at the edge of copper *versus* lead bullets presented a significant difference (*p* = 0.002) and a gap between the CT numbers of copper and lead bullets and was successfully applied for the decedents.

**Conclusions:**

This study presents a viable method for distinguishing copper and lead bullets *in situ* via CT and highlights the potential pitfalls of incorrect classifications.

## Key points

- Computed tomography (CT) numbers are not reliable for distinguishing copper from lead bullets.
- The dual-energy index (DEI), representing the ratio between the CT numbers of two energies, is more reliable for classifying those bullets.
- The ratio of maximum CT numbers (DEI_max_) was suitable for classifications.
- Using the 120/140 DEI_max_ from CT numbers of bullets’ edges is recommended.

## Background

Computed tomography (CT) allows identification of the location of a lodged projectile and detection of gunshot residues indicating a contact shot [1, 2]. Ballistic experts examine bullets secured at a crime scene or removed from a body, and laboratory analysis of the deposits from an entrance wound can provide information on the bullet used [3–5]. The *in situ* identification of a bullet can be particularly interesting in forensic investigations [6–8] when a lodged bullet will not be removed from the patient, for example, to avoid the risk of neural damage due to an intervention [9]. The feasibility of visual assessment or caliber measurement of lodged bullets using CT was assessed on real shooting victims in postmortem studies [10–12]. The authors of these studies concluded that visual assessments and caliber measurements on CT are often impeded or infeasible since lodged bullets are frequently heavily deformed or fragmented [13–16]. Therefore, a method that is less dependent on the intactness of a lodged bullet is desired.

Dual-energy-based material differentiation of bullets using clinical CT scanners was assessed in *ex situ*, animal cadaver, and phantom studies [17–19]. The x-ray attenuation of a material can be measured as CT numbers (Hounsfield unit (HU) values) within a defined region of interest (ROI) including several pixels/voxels. Calculation of a bullet’s dual-energy index (DEI) from CT numbers measured at two different energy levels (dual-energy) was recently presented as a robust method for distinction between intact bullets composed of copper (and zinc) and those composed of lead, which were manually inserted into animal cadaver models [18]. Repeated single-energy CT scans at different energy levels were performed instead of actual dual-energy CT scans, since only single-energy CT scans allowed for reconstructions that enabled CT number measurements beyond the standard range of HU values [18]. Therefore, this DEI method is feasible using any standard CT scanner, but it causes additional radiation exposure. However, previous *ex situ* studies on foreign bodies demonstrated significantly different CT numbers between copper or brass (a copper-zinc alloy) specimens and lead specimens at a single energy of 130 kVp [20, 21]; thus, the DEI method might be superfluous for the distinction between copper and lead bullets. Therefore, further investigation of the x-ray attenuation characteristics of copper and lead bullets and exploration of the potential benefit of using the DEI compared to using CT numbers for the differentiation of these two types of bullets were deemed necessary.

This study aimed (1) to investigate the need for two CT scans at different energy levels for the material differentiation of lodged bullets composed of copper and lead in an animal cadaver study and (2) to present a reliable and valid method for differentiating between these two types of frequently encountered bullets using clinical CT scans.

## Methods

No animals were killed for the scientific purposes of this study. The animal models used in this study were obtained from an institute of veterinary pathology. Fresh cadavers were used as an addition to another study with ethical approval and are in accordance with the 3Rs (replacement, reduction, and refinement)—the guiding principles for the ethical use of animals in science. Additional ethical approval for using these animal cadavers was not required. Parts of this study were performed with human cadavers. Ethical approval was waived by the responsible ethics committee of the Canton of Zurich (waiver number: 2015-0686). This article does not contain any studies with (living) human participants.

### Animal cadaver study and real forensic cases

**Fig. 1:**
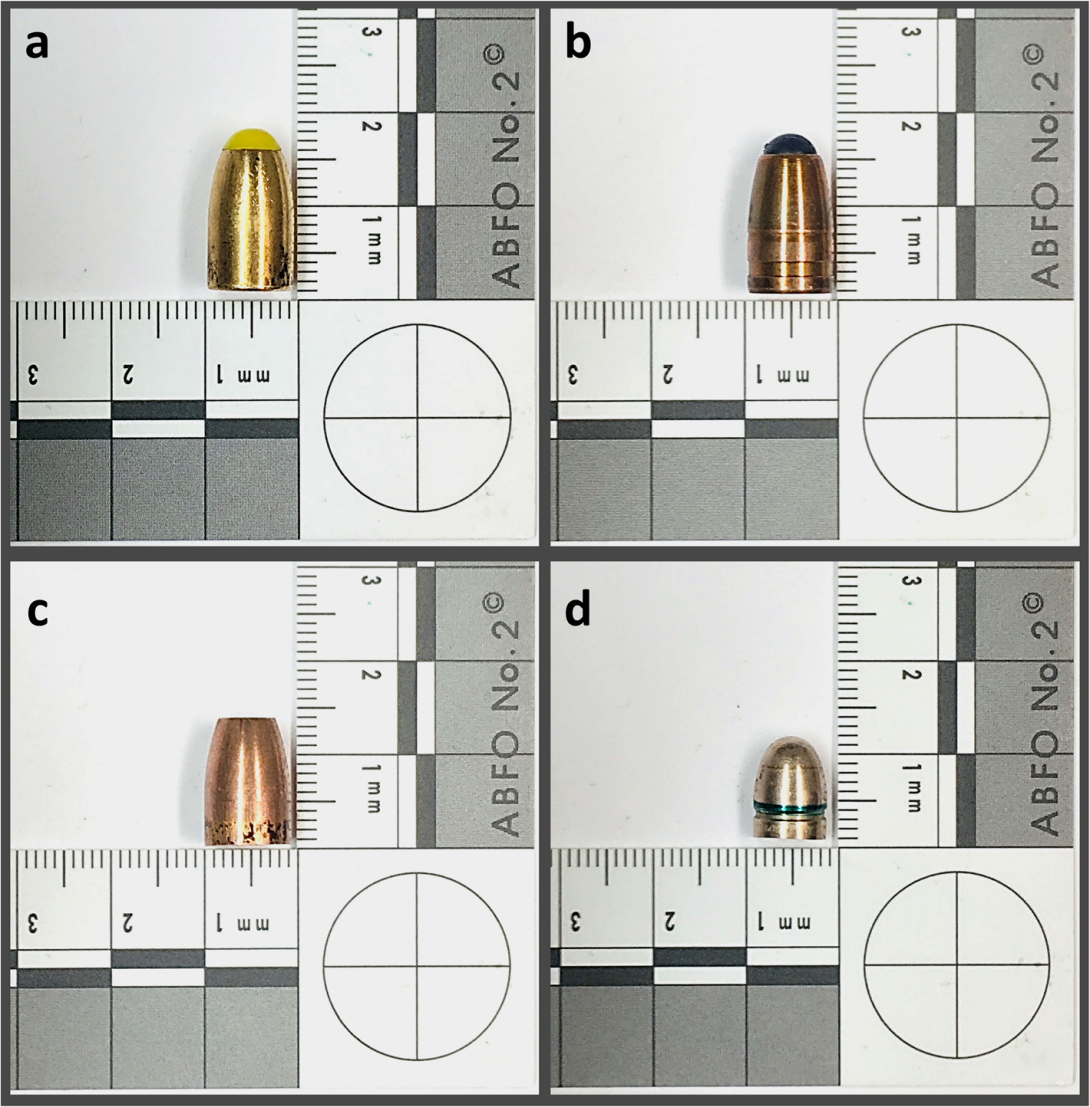
*Action 4* (**a**), *QD-PEP* (**b**), *Hydra-Shok* (**c**), and *7.65 Browning* (**d**) bullets were fired into animal cadaver models at a dedicated shooting range. Then, computed tomography scans of the animal cadaver models with lodged copper bullets (**a**, **b**) and lodged lead bullets (**c**, **d**) were performed

Bullets (*n* = 12) from four different types of ammunition were selected for this study (*Action 4*, *n* = 3; *QD-PEP*, *n* = 3; *Hydra-Shok*, *n* = 3; *7.65 Browning*, *n* = 3) (Fig. 1). The bullets were divided into two groups according to their core materials. One group (copper group, *n* = 6) included the unjacketed *Action 4* and *QD-PEP* bullets, which are composed of copper. These solid copper bullets are deformation bullets that were developed for law enforcement units. The other group (lead group, *n* = 6) included the *Hydra-Shok* and *7.65 Browning* bullets, which are frequently encountered lead bullets with jackets composed of copper-zinc alloys (copper/zinc). The *Hydra-Shok* bullet is a semi-jacketed hollow-point (deformation) bullet, while *7.65 Browning* bullets (which are also referred to as *.32 ACP* bullets) are full metal-jacketed bullets. From each type of ammunition, three bullets were fired into animal cadaver models at a dedicated shooting range. Sheep legs were used as a substitute for human tissue. The shootings were performed by a ballistics expert from a forensics institute. After shooting, each sheep leg was scanned by CT.

Additionally, the distinction between copper and lead bullets was assessed in real forensic cases with fatal gunshot wounds and lodged bullets. The decedents (*n* = 15) underwent postmortem imaging as part of forensic judicial investigations. Ethical approval was waived by the responsible ethics committee. The bullets were removed during autopsy and identified by the forensics institute. Before the bullets were removed, the decedents underwent a CT examination using the same scanner used for the animal cadaver study. The CT scan protocol from the animal cadaver study was used. Decedents with a lodged *Action 4* copper bullet (*n* = 3) and decedents with a lodged *.22 LR* lead bullet (*n* = 3) were selected for this study.

### Scan protocol

Repeated CT scans using energy levels of 80, 100, 120, and 140 kVp were performed using a standard medical 128-slice CT scanner (SOMATOM Definition Flash, Siemens Healthcare GmbH, Forchheim, Germany). The tube current was adjusted to gain an almost equal volume CT dose index of 9 mGy at each energy level, which provides equivalent image noise. A standard pitch of 0.6 was used. The raw data were reconstructed using standard filtered back projection with a hard kernel (B70), a slice thickness of 1.5 mm, and a field of view of 140 × 140 mm (reconstruction matrix, 512 × 512; in-plane voxel size, 0.27 × 0.27 mm). Reconstructions were calculated in an extended CT scale (ECTS) to allow measurements beyond the standard range of HU values [22].

### ROI measurements, CT numbers, and the dual-energy index

**Fig. 2:**
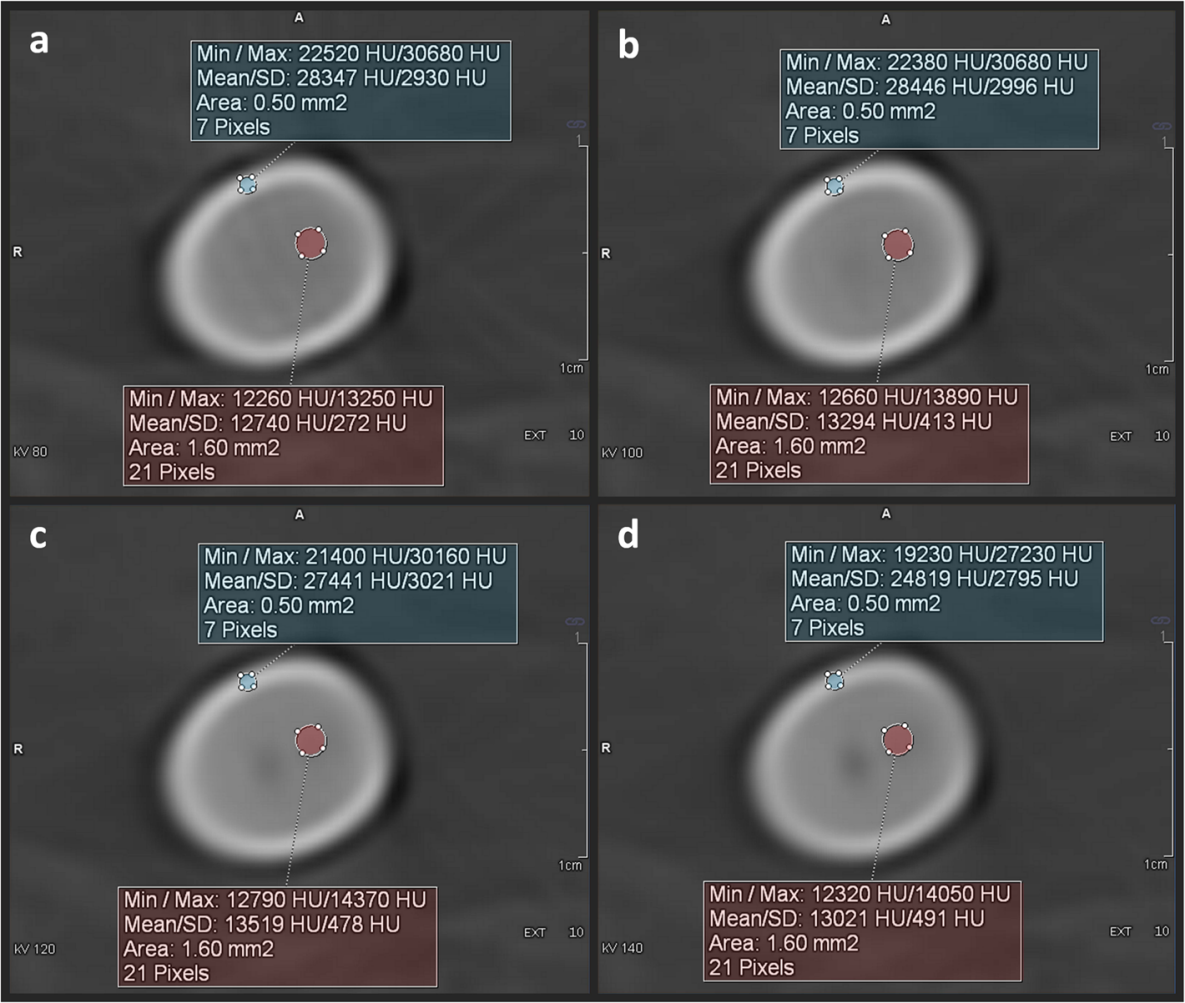
Cross sections of a lodged *Action 4* copper bullet at different energy levels indicated in kilo-voltage-peaks (kVp) (**a**, 80 kVp; **b**, 100 kVp; **c**, 120 kVp; **d**, 140 kVp). Region-of-interest-based measurements were carried out in the hyperdense ring at the edge (area 0.5 mm^2^, highlighted in blue) and in the centre of the bullet (area 1.6 mm^2^, highlighted in red). Each pixel (*i.e.,* voxel) contains a single CT number. The measurements indicate the mean computed tomography (CT) number, the standard deviation (SD), the minimum CT number, and the maximum CT number of all pixels within the ROI. The CT numbers (*i.e.,* the x-ray attenuation values) are influenced by the energy level. CT numbers obtained from two different energy levels can be used to calculate the dual-energy index (DEI), which represents the ratio of the CT numbers of the two energy levels

CT numbers were measured in a defined ROI at 80, 100, 120, and 140 kVp (Fig. 2). To assure identical ROI placement, the datasets were displayed side by side in a multiplanar reconstruction view using dedicated software (MM Reading, syngo.via, Version VB10B HF03, Siemens Healthcare GmbH, Forchheim, Germany) [23]. The software enables the mean and maximum CT numbers to be measured within an ROI at the exact same position on all four datasets with different energy levels. ROI circles were drawn at the centre (ROI: 1.6 mm^2^) and edge (ROI: 0.5 mm^2^) of the lodged bullet. Measurements were taken separately at these two positions to demonstrate the influence of the bullet’s caliber. For each bullet, six ROIs were positioned at different slices, *i.e.,* different levels within the bullet or its fragments for the core and edge measurements (ROIs per bullet: core, *n* = 6; edge, *n* = 6). An ROI was repositioned on a new slice if the upper limit of 30,710 HU was displayed as the maximum CT number. The DEI was calculated for dual-energy pairs of 80/100 kVp, 80/120 kVp, 80/140 kVp, 100/120 kVp, 100/140 kVp, and 120/140 kVp using the mean CT numbers (DEI_mean_) and the maximum CT numbers (DEI_max_) from the ROI measurements at the centre and edge of the lodged bullet. The following formula [24] was used to calculate the DEI:

$$ DEI=\frac{x_{\mathrm{low}}-{x}_{\mathrm{high}}}{x_{\mathrm{low}}+{x}_{\mathrm{high}}+2000} $$

The variable *x*_low_ represents the CT number measured at the lower energy level of the individual dual-energy pair, while *x*_high_ is the CT number measured at the higher energy level.

### Distinction between copper and lead bullets

The difference between using CT numbers from a single energy and the DEI for the distinction between copper and lead bullets within the animal cadaver models was assessed by considering the energy level, the use of the mean and maximum CT numbers, and the ROI position (core or edge). The two groups of bullets were compared using statistical analysis, standard deviations, and data overlap. Finally, the most suitable method with the lowest standard deviations and the smallest data overlap was applied and assessed in real forensic cases.

### Statistical analysis and data overlap calculations

The overall mean values of the mean CT numbers, of the maximum CT numbers, and of the DEIs of each bullet were used for statistical analysis. The Shapiro-Wilk test was used to determine whether the data were normally distributed. The *t* test was used for normally distributed data, and the Mann-Whitney *U* test was used for non-normally distributed data to reveal statistically significant differences between the two groups of bullets (significance level, *p* < 0.05). The statistical analyses were performed using the Statistical Package for the Social Sciences (SPSS, International Business Machines Corporation, IBM, Armonk, NY, USA).

## Results

### Animal cadaver study

**Fig. 3:**
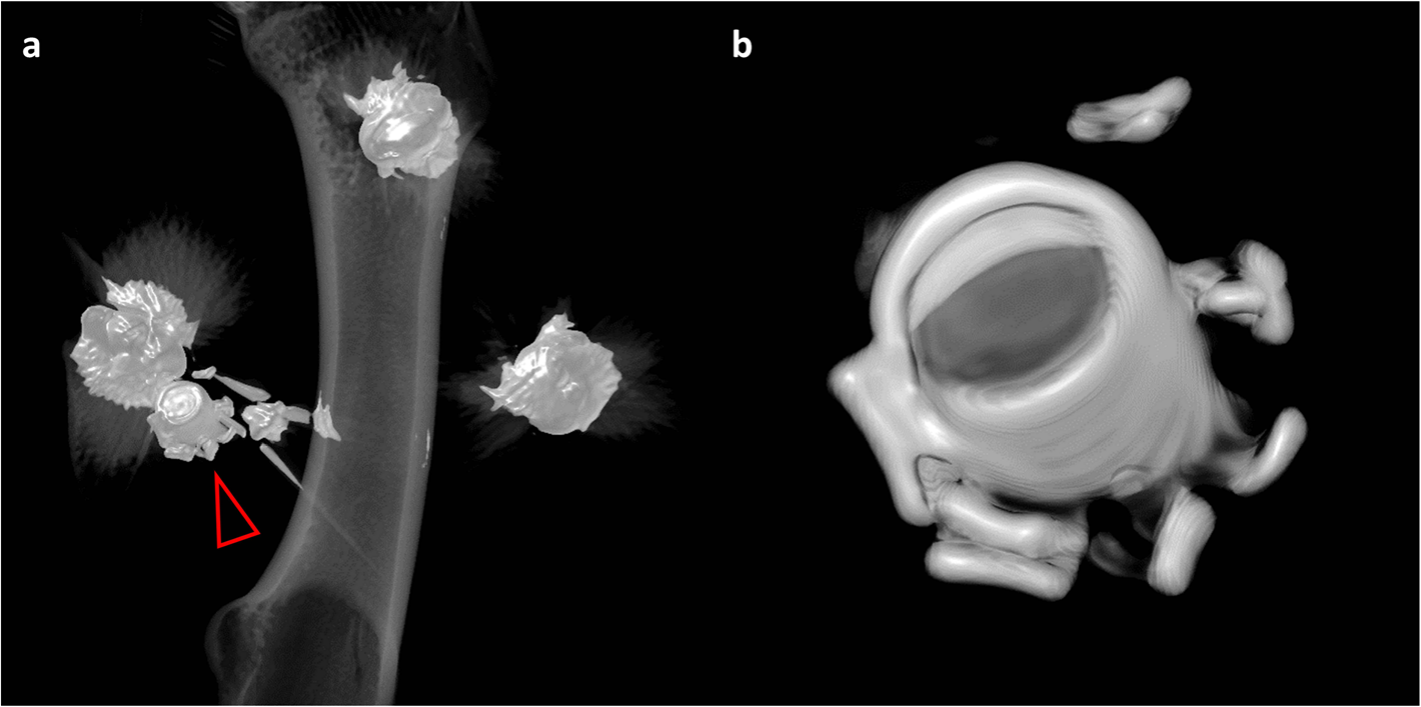
Volume rendering of the *Hydra-Shok* bullets within the animal cadaver model obtained from the computed tomography scan with 140 kVp (**a**). The copper/zinc jacket (**a**, arrowhead; **b**) of one of the *Hydra-Shok* bullets (*Hydra-Shok* number 2) was separated from the lead core of the bullet (**a**, arrowhead). All *Hydra-Shok* bullets were heavily deformed; however, they did not hit the femoral bone as no osseous fractures were detected. Metal artifacts are visible as streaks in the panel **a**, but these streaks disappeared when a window that could precisely visualise the metallic object was selected (**b**)

All bullets were deformed, and the lead bullets were partially fragmented. If a bullet was fragmented, ROI measurements were conducted on the largest main fragment. One of the lead bullets was separated from its jacket, which was composed of a copper-zinc alloy (Fig. 3). This particular jacket was used to further examine the final method applied on real forensic cases in this study.

### Differences in CT numbers from single energies

**Fig. 4:**
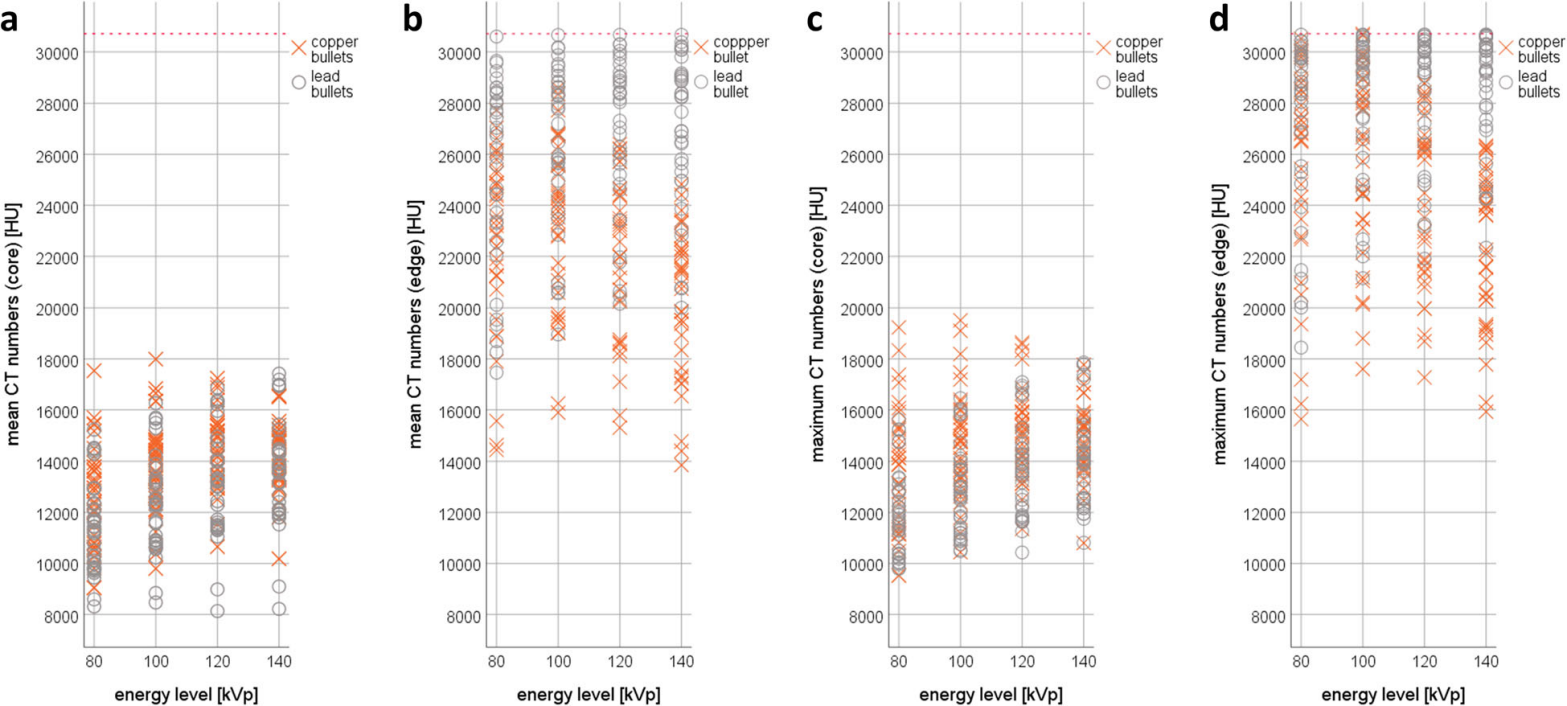
Mean computed tomography (CT) numbers obtained from the cores (**a**) and the edges (**b**) of the bullets and the maximum CT numbers obtained from the cores (**c**) and the edges (**d**) of the bullets. The CT numbers of these two types of bullets demonstrate a strong overlap. The CT numbers from the cores were much lower than those from the edges. The red dashed line indicates the upper limit of the extended CT scale (ECTS)

The mean and maximum CT numbers of a total of 576 ROI measurements are illustrated in Fig. 4. Table 1 lists the overall mean values and standard deviations, minimum and maximum values, and the statistical analysis of the mean and maximum CT numbers for the two groups at all four energy levels from the ROI measurements at the core and the edge of the lodged bullets.

**Table 1:** CT numbers of copper and lead bullets

Measurements			Mean ± standard deviation		Min	Max	Min	Max	SW test	*t* test
CT number	ROI position	Energy (kVp)	Copper bullets	Lead bullets	Copper bullets		Lead bullets		*p* value	*p* value
Mean	Core	80	12,584 ± 1,951	11,406 ± 1,724	9,041	17,535	8,312	15,227	0.448	0.836
		100	13,862 ± 1,729	12,315 ± 1,867	9,798	17,988	8,469	16,266	0.403	0.399
		120	14,462 ± 1,435	12,996 ± 1,959	10,654	17,243	8,133	16,869	0.610	0.155
		140	14,197 ± 1,300	13,514 ± 2,048	10,180	16,571	8,217	17,419	0.948	0.169
Max	Core	80	13,649 ± 2,368	11,945 ± 1,645	9,520	19,230	9,790	15,630	0.457	0.714
		100	14,734 ± 2,004	12,848 ± 1,692	10,440	19,510	10,480	16,460	0.609	0.629
		120	15,186 ± 1,639	13,586 ± 1,754	11,350	18,640	10,430	17,,090	0.463	0.347
		140	14,820 ± 1,389	14,054 ± 1,838	10,800	17,770	10,810	17,860	0.397	0.275
Mean	Edge	80	22,708 ± 3,360	25,488 ± 3,617	14,448	27,730	17,461	30,598	0.991	0.423
		100	23,295 ± 3,212	26,412 ± 3,136	15,897	28,579	18,956	30,660	0.975	0.888
		120	21,976 ± 3,036	26,751 ± 2,995	15,304	26,394	20,160	30,668	0.896	0.727
		140	20,272 ± 2,840	26,976 ± 2,880	13,854	24,828	20,000	30,665	0.460	0.532
Max	Edge	80	25,854 ± 4,045	27,251 ± 3,395	15,640	30,510	18,440	30,670	0.364	0.421
		100	25,934 ± 3,582	28,068 ± 2,731	17,600	30,700	21,160	30,680	0.734	0.733
		120	24,277 ± 3,248	28,340 ± 2,478	17,270	28,780	21,910	30,680	0.448	0.818
		140	22,290 ± 2,926	28,628 ± 2,226	15,930	26,340	22,340	30,680	0.168	0.655

*Min* Minimum value, *Max* Maximum value, *ROI* Region of interest; *SW test* Shapiro-Wilk test

At all four energy levels, the mean values of the (mean and maximum) CT numbers obtained from core measurements were higher in the copper group than in the lead group, while the mean values from the edge measurements exhibited the opposite relationship (*i.e.,* the (mean and maximum) CT numbers obtained from core measurements were higher in the lead group than in the copper group). The standard deviations decreased with an increase in the energy level in the copper group; the same phenomenon occurred for the edge measurements in the lead group, while in the lead core, the standard deviations increased with an increase in the energy level. Furthermore, the (mean and maximum) CT numbers obtained from lead bullets increased with an increase in the energy level from 80 to 140 kVp. However, the CT numbers measured in the copper bullets presented an increase only from 80 to 120 kVp in the core and from 80 to 100 kVp at the edge. A peak occurred for the CT numbers at 120 kVp (core) and at 100 kVp (edge), and the CT numbers decreased at higher energy levels. The Shapiro-Wilk test indicated that the mean and maximum CT numbers were normally distributed; therefore, the *t* test was applied. The *t* tests revealed no statistically significant differences between the CT numbers of copper and lead bullets.

### Differences in the dual-energy index

**Fig. 5:**
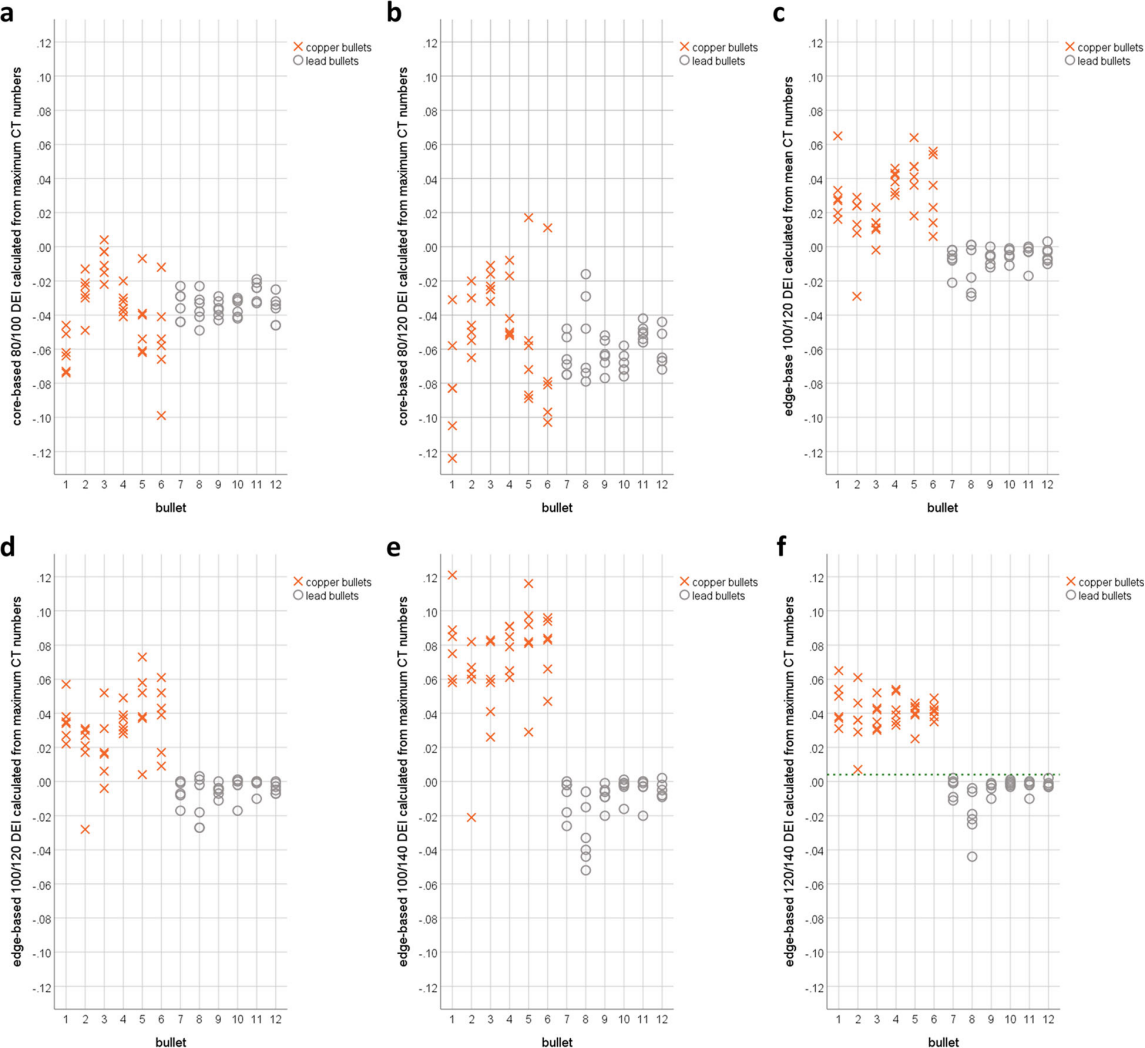
The core-based 80/100 dual-energy index (DEI)_max_ (**a**), the core-based 80/120 DEI_max_ (**b**), the edge-based 100/120 DEI_mean_ (**c**), the edge-based 100/120 DEI_max_ (**d**), the edge-based 120/140 DEI_max_ (**e**), and the edge-based 120/140 DEI_max_ (**f**) yielded statistically significant differences between the two groups of bullets (*x*-axis: *Action 4* bullets, 1-3; *QD-PEP* bullets, 4-6; *Hydra-Shok* bullets, 7-9; *7.65 Browning* bullets, 10-12). While large overlaps are visible for core measurements, the copper group visibly differed from the lead group in terms of the DEI calculated from edge measurements. Only the edge-based 120/140 DEI_max_ resulted in a clear dividing line between the two groups of bullets (green dashed line). The *Hydra-Shok no. 2* (bullet number 8 on the *x*-axis), which was separated from its jacket, yielded lower values than all other copper/zinc-jacketed lead bullets.

Table 2 lists the overall mean values and standard deviations or median values and interquartile ranges, minimum and maximum values, and the statistical analysis of the DEIs of each group calculated from core-based and edge-based CT numbers for all six dual-energy pairs. The copper group demonstrated higher DEIs than the lead group except for the 80/100 and 80/120 DEI_mean_ values and the 80/100 DEI_max_ value based on core measurements. In the core, statistically significant differences between copper and lead bullets were detected for the 80/100 and 80/120 DEI_max_ values (each, *p* = 0.002). Concerning edge measurements, a statistically significant difference was detected for the 100/120 DEI_mean_ value and 100/120, 100/140, and 120/140 DEI_max_ values (each, *p* = 0.002). The DEIs of the lead bullets that differed significantly from the copper bullets presented small standard deviations (range, ± 0.008 to ± 0.014) or interquartile ranges (range, 0.005−0.015), while those of the copper bullets presented larger standard deviations (range, ± 0.023 to ± 0.036) or interquartile ranges (range, 0.024-0.028) except for the 120/140 DEI_max_. The edge-based 120/140 DEI_max_ presented small interquartile ranges in both groups of bullets (copper bullets, 0.011; lead bullets, 0.005) and a gap between the calculated DEIs of the two groups (Fig. 5); therefore, the edge-based 120/140 DEI_max_ was deemed the most appropriate for distinguishing between copper and lead bullets. A boundary at 0.004 was identified between the two groups.

**Table 2:** Dual-energy indexes for copper and lead bullets

Measurements			Mean ± standard deviation or median (interquartile range)		Min	Max	Min	Max	SW test	*t* test or MW *U* test
CT number	ROI position	Dual energy (kVp/kVp)	Copper bullets	Lead bullets	Copper bullets		Lead bullets		*p* value	*p* value
Mean	Core	80/100	-0.047 ± 0.020	-0.035 ± 0.009	-0.091	-0.011	-0.051	-0.008	0.514	0.090
		80/120	-0.068 ± 0.031	-0.060 ± 0.016	-0.140	0.014	-0.081	0.010	0.351	0.070
		80/140	-0.059 ± 0.039	-0.078 ± 0.020	-0.132	0.056	-0.103	0.005	0.693	0.080
		100/120	-0.021 ± 0.015	-0.025 ± 0.009	-0.050	0.027	-0.039	0.018	0.858	0.259
		100/140	-0.013 ± 0.024	-0.043 ± 0.013	-0.059	0.068	-0.056	0.013	0.516	0.323
		120/140	0.008 ± 0.011	-0.018 ± 0.006	-0.011	0.042	-0.029	-0.005	0.104	0.264
Max	Core	80/100	-0.038 ± 0.023	-0.034 ± 0.008	-0.099	0.004	-0.049	-0.019	0.405	0.014*
		80/120	-0.054 ± 0.036	-0.060 ± 0.014	-0.139	0.017	-0.079	-0.016	0.880	0.016*
		80/140	-0.042 (0.074)	-0.076 (0.015)	-0.122	0.068	-0.098	-0.021	0.014*	0.065
		100/120	-0.016 ± 0.018	-0.026 ± 0.011	-0.051	0.023	-0.048	0.007	0.731	0.612
		100/140	-0.005 ± 0.027	-0.042 ± 0.011	-0.050	0.075	-0.055	0.002	0.233	0.272
		120/140	0.011 ± 0.013	-0.016 ± 0.009	-0.013	0.051	-0.034	0.003	0.154	0.521
Mean	Edge	80/100	-0.013 ± 0.031	-0.019 ± 0.022	-0.129	0.052	-0.082	0.047	0.150	0.829
		80/120	0.015 ± 0.046	-0.025 ± 0.024	-0.158	0.111	-0.109	0.027	0.228	0.278
		80/140	0.053 ± 0.050	-0.030 ± 0.031	-0.141	0.178	-0.131	0.016	0.481	0.675
		100/120	0.028 (0.028)	-0.005 (0.008)	-0.029	0.065	-0.029	0.003	0.037*	0.002*
		100/140	0.066 ± 0.025	-0.011 ± 0.014	-0.012	0.134	-0.050	0.017	0.114	0.232
		120/140	0.039 ± 0.011	-0.004 ± 0.010	0.012	0.069	-0.037	0.025	0.062	0.418
Max	Edge	80/100	-0.004 (0.034)	-0.007 (0.016)	-0.103	0.063	-0.084	0.000	0.027*	0.065
		80/120	0.028 ± 0.045	-0.021 ± 0.024	-0.131	0.094	-0.111	0.000	0.623	0.649
		80/140	0.069 ± 0.050	-0.026 ± 0.031	-0.124	0.158	-0.136	0.000	0.276	0.952
		100/120	0.032 (0.024)	-0.002 (0.007)	-0.028	0.073	-0.027	0.003	0.039*	0.002*
		100/140	0.080 (0.028)	-0.006 (0.015)	-0.021	0.121	-0.052	0.002	0.017*	0.002*
		120/140	0.040 (0.011)	-0.002 (0.005)	0.007	0.065	-0.044	0.002	0.012*	0.002*

*Min* Minimum value, *Max* Maximum value, *ROI* Region of interest; *SW test* Shapiro-Wilk test; *MW U test* Mann-Whitney *U* test

*Statistically significant values

The 120/140 DEI_max_ was also calculated for the jacket composed of copper (and zinc) that was separated from the lead core after entering the animal cadaver (Fig. 3). The jacket yielded a 120/140 DEI_max_ above the threshold (mean value, 0.064; range, 0.051–0.072), which was also observed for all solid copper bullets. Consequently, the separated jacket clearly differed from its initial lead core and from all (still) jacketed lead bullets in the 120/140 DEI_max_. Interestingly, the *Hydra-Shok* no. 2 bullet, the “unjacketed” lead bullet, presented lower DEIs than the other lead bullets (Fig. 5).

### Real forensic cases

**Fig. 6:**
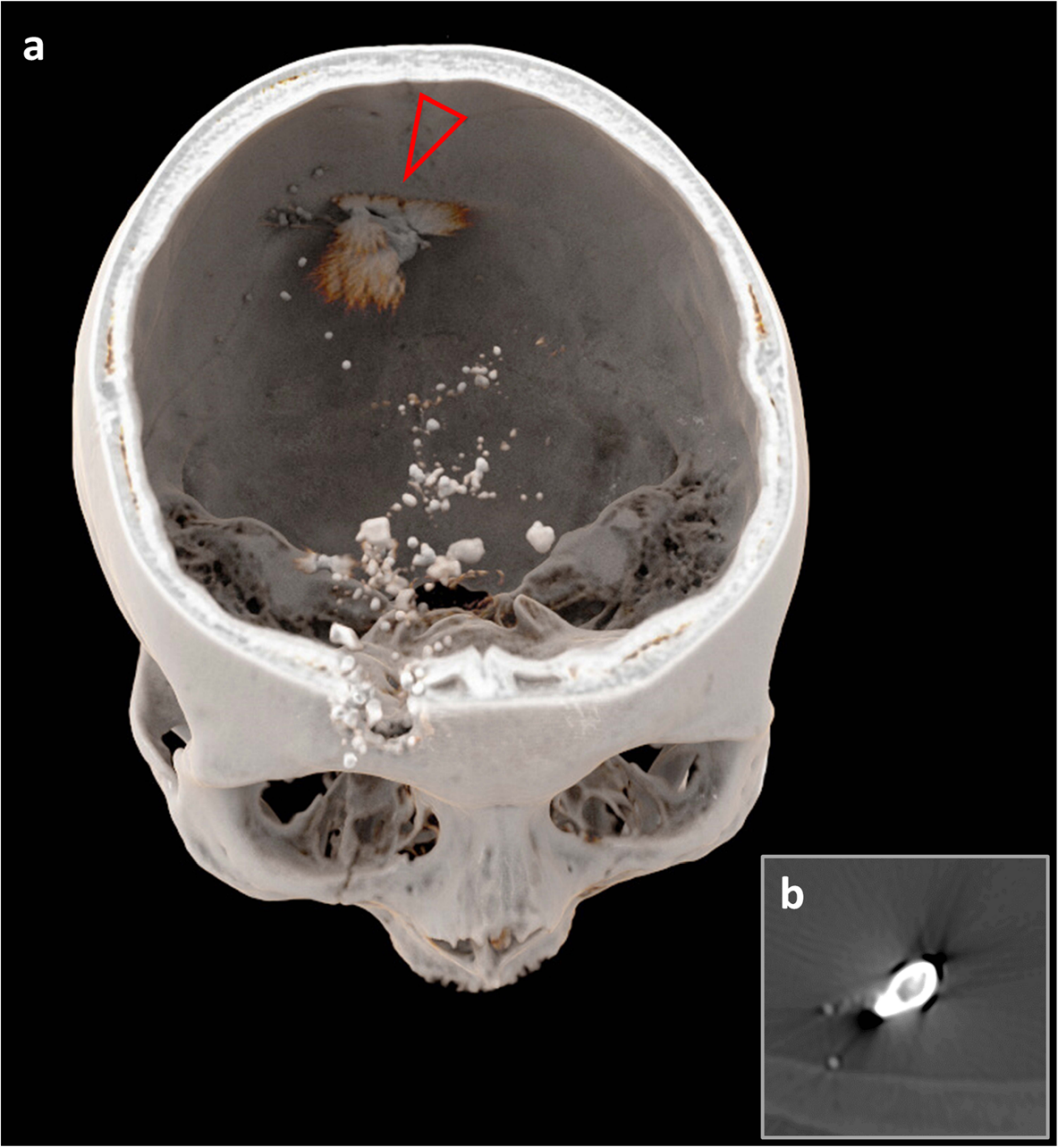
Cinematic rendering of a cerebral non-perforating gunshot wound (case 1) obtained from a computed tomography (CT) examination of the decedent’s head at 120 kVp (**a**). The entrance wound is located on the left side of the frontal bone. Numerous bone fragments and tiny metal fragments are scattered along the bullet path. The bullet fragment is lodged in the occipital lobe above the posterior cranial fossa (**a**, arrowhead). Visual identification of the bullet was not feasible via CT due to its severe deformation (**b**)

**Fig. 7:**
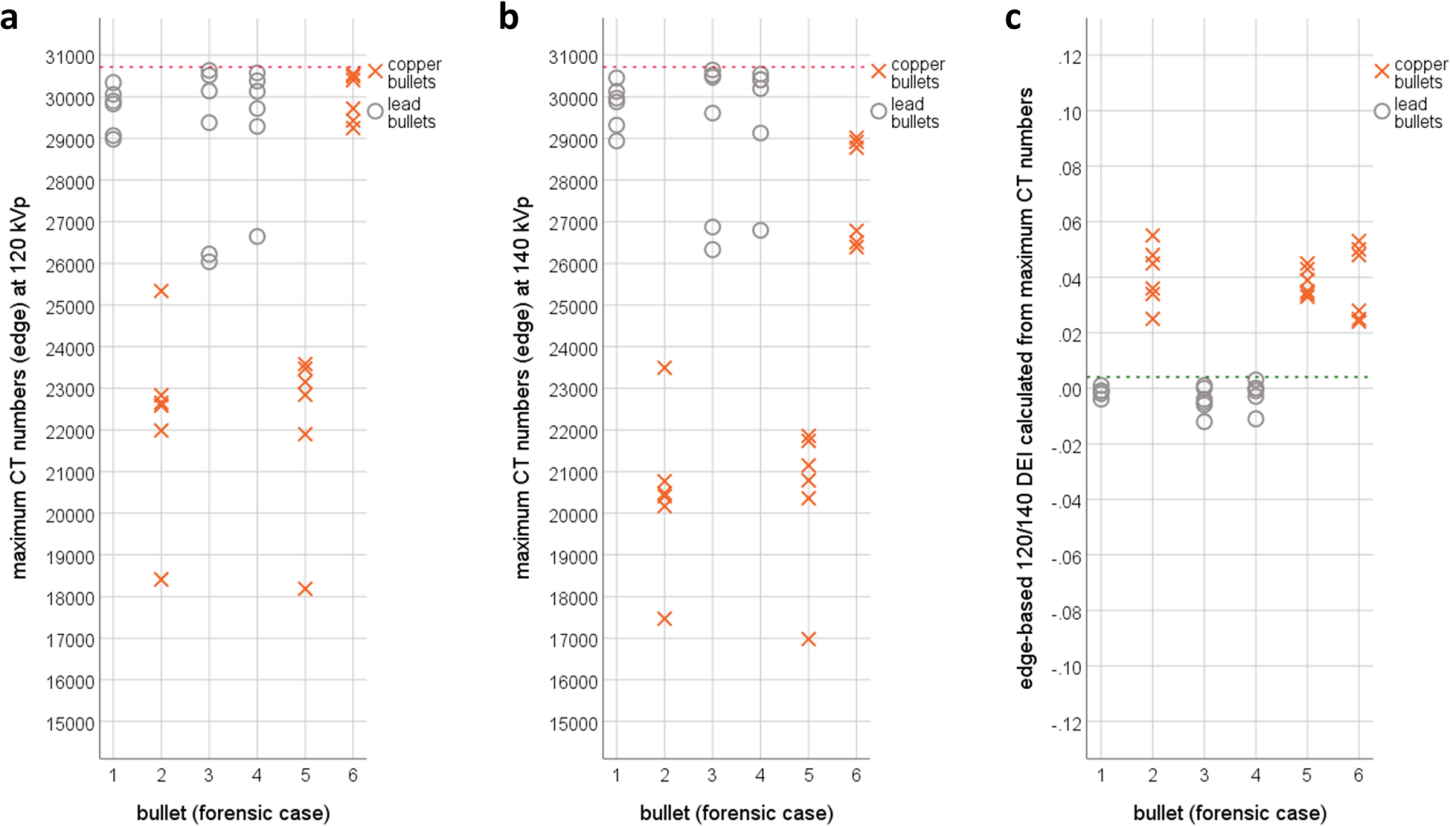
Maximum computed tomography (CT) numbers from the edge at 120 kVp (**a**) and at 140 kVp (**b**) and the 120/140 DEI_max_ calculated from CT numbers obtained from edge measurements (**c**). Only the lodged *Action 4* copper bullet in cases 2 and 5 demonstrated far lower maximum CT numbers than the lodged *.22 LR* lead bullets in cases 1, 3, and 4. However, the *Action 4* copper bullet in case 6 did not differ from the lead bullets at either 120 kVp (**a**) or 140 kVp (**b**). At 120 kVp, for the *Action 4* copper bullet, values very close to the upper limit of the extended CT scale (ECTS) represented as red-dashed line were reached. In contrast, all *Action 4* copper bullets clearly differed from the *.22 LR* lead bullets in terms of the dual-energy index (DEI) and obtain DEIs above the threshold of 0.004 represented as green dashed line defined by the results of the animal cadaver study. Even the *Action 4* copper bullet in case 6 clearly differed from the lead bullets in terms of the DEI, which was calculated from CT numbers that were not obviously different from those of lead bullets at 120 and 140 kVp.

**Fig. 8:**
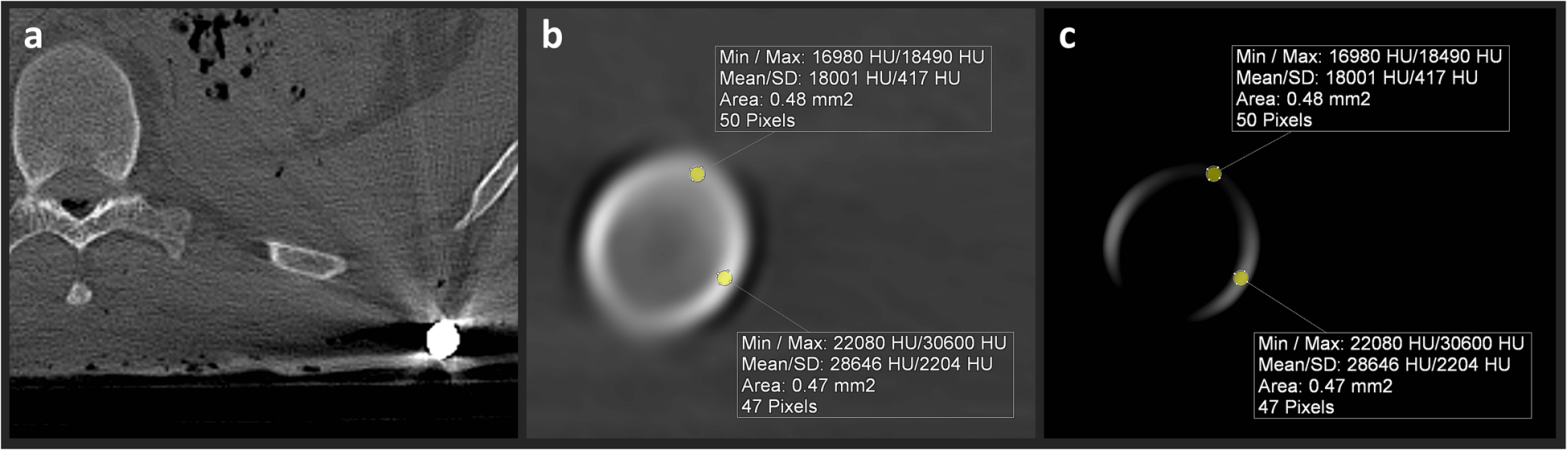
The *Action 4* copper bullet in case 6 was lodged in the dorsal muscles (**a**). The computed tomography numbers at 120 kVp varied from those that are consistent with copper bullets (**b**, **c**: 18,490 HU) to those that are consistent with lead bullets (**a**, **b**: 30,600 HU). A high window centre can be used to illustrate unevenly distributed beam hardening along the hyperdense ring at the edge of the bullet (**c**). Since the beam hardening artifact is pronounced in the diagonal vertical direction of the x-ray beam, the vertebral bone and x-ray scattering may have affected the intensity of beam hardening at the edge of this particular bullet

Similar to the animal cadavers, all three *Action 4* copper bullets lodged in decedents were deformed, while all three unjacketed *.22 LR* lead bullets were deformed and partially fragmented. The bullets were located in the cranium (cases 1, 2, and 4), the dorsal muscles (cases 3 and 6), and the muscles of the upper arm (case 5). The lead bullets fragmented into several pieces and tiny metal fragments were scattered along the wound channel. The fragments did not allow visual classification or caliber measurements (Fig. 6). According to the results of the animal cadaver study, the edge-based 120/140 DEI_max_ was applied for the decedents. Since the CT numbers at 120 kVp and 140 kVp had to be measured for calculation of the DEI, these CT numbers were also compared between the individual cases. At both energy levels, only two of the three *Action 4* copper bullets (cases 2 and 5) presented lower CT numbers than the *.22 LR* lead bullets in all other cases, while the *Action 4* copper bullet in case 6 was not distinguishable from the lead bullets using the CT numbers (Fig. 7a and b). However, the 120/140 DEI_max_ allowed a clear distinction between copper and lead bullets (Fig. 7c). All lead bullets presented DEI values below the threshold of 0.004, while all copper bullets had DEI values far above this threshold. The *Action 4* bullet in case 6, which did not differ from lead bullets according to its CT numbers, was located approximately 12 cm from the 10th thoracic vertebra. A closer look at this particular copper bullet revealed that beam hardening was very unevenly distributed along the hyperdense ring at the edge of the bullet (Fig. 8).

## Discussion

This study highlights some important factors that must be considered for the differentiation between copper and lead bullets according to their metallic components using CT. At 80, 100, 120, and 140 kVp, the CT numbers of copper bullets did not significantly differ from those of lead bullets, and using CT numbers alone can lead to false classifications. The ratio between CT numbers at two different energy levels indicated by the DEI is more suitable to distinguish copper from lead bullets. For this ratio, the maximum CT numbers appeared to be more appropriate than the mean CT numbers. In the core, only the CT number ratios between low energies (80/100 and 80/120 DEI_max_) presented significant differences but these CT number ratios presented also large data overlaps and large standard deviations for the copper bullets. In contrast, in the edge, only the CT number ratios between high energies presented significant differences (100/120, 100/140, and 120/140 DEI_max_). Only the 120/140 DEI_max_ obtained from edge measurements exhibited small standard deviations for both groups of bullets and a gap between the data of both groups. The edge-based 120/140 DEI_max_ was successfully introduced into postmortem imaging of deceased gunshot victims.

Beam hardening occurs at the edge of a bullet or its fragment [18, 25]; thus, the measured CT numbers are not “real”. However, while this physical effect barely affects the DEI, it considerably affects the CT number, as demonstrated in case 6 of the real forensic cases, where the CT numbers were unexpectedly high for a copper bullet. Nonetheless, the DEI calculated from those unexpectedly high CT numbers still allowed clear classification of the bullet due to the slight decrease in the maximum CT numbers from 120 to 140 kVp. An increase or a decrease in CT numbers over all four energy levels is related to the atomic numbers (*Z*) and the *K*-edge energies of the individual metals (copper, *Z* = 29; *K*-edge = 8.9 keV; lead, *Z* = 82; *K*-edge = 88.0 keV) and the photoelectric effect [18]. Differentiation between bullets composed of metallic components with atomic numbers that are close together in the periodic table might be considerably more challenging using the DEI-based approach.

Maximum CT numbers are usually not used since they are strongly affected by quantum and image noise. Therefore, using the same scanning and reconstruction parameters as well as the same volume CT dose index is important for repeated scans with two different energies. Complying with these conditions, the ratio between maximum CT numbers within the same ROI from two different energy levels can be a robust indicator for the attenuation characteristics of metallic objects.

Although the ROIs at the edge of the jacketed lead bullets very likely included some pixels/voxels located in the bullets’ jackets, which are composed of copper-zinc alloy, the material of the jacket did not affect the identification of lead bullets. The DEIs of the jackets of lead bullets in the animal cadaver study did not noticeably differ from those of the unjacketed *.22 LR* bullets in the real forensic cases. Lead presents very high CT numbers; thus, the CT numbers of the less radiopaque metal in the jacket hardly affects the mean or maximum CT numbers obtained from ROI measurements. However, a jacket composed of copper-zinc alloy that is separated from the bullet, which occurred once in this study, can be differentiated from its unjacketed lead core or other jacketed lead bullets.

Core measurements are considered unreliable for differentiating between copper and lead bullets since large data overlaps were calculated and large standard deviations were observed for the copper bullets. Similar to CT numbers measured at the edge, CT numbers measured in the centre are not “real”. Previous studies on material differentiation [20, 21, 26] used the mean CT numbers from the centre of the objects to distinguish between metallic foreign bodies. The authors pointed out that CT numbers must be measured far from near-surface regions to ensure reliable HU values and to avoid partial volume effects [20, 21, 26]. However, the CT number of a metallic object strongly decreases with the x-ray penetration depth. This cupping effect, which increases with the diameter of a bullet, was shown for intact bullets in a previous *ex situ* study on intact bullets [25]. Additionally, photon starvation increases with the size of a radiopaque object, indicating that the detector receives noisier information regarding the x-ray attenuation at the centre of the material. Consequently, the CT numbers obtained from a radiopaque material vary depending on the ROI position and the ROI size.

Some limitations of this study should be noted. First, only bullets composed of copper or lead were investigated. However, these are the most frequently encountered types of bullets [27, 28]. Other metallic components, such as steel, bismuth, and tungsten, are much less often used for the bullet core, while steel is more frequently used for the jackets of bullets. To distinguish ferromagnetic steel-jacketed bullets from non-ferromagnetic non-steel-jacketed bullets, different studies yielded contradictory results [17, 29] since the type of metal used for the core of the bullet (usually copper or lead) was not considered [18, 29]. Notably, some bullets have different core metals at the point than at the body. Second, only a small number of bullets were used in this study, consequently minimising the statistical power of the results. Additionally, the ROIs at the edges of the bullets contained only a small number of pixels (see Fig. 2). Despite the small number of bullets used in the animal cadaver study and the small number of pixels in the ROIs, the selected 120/140 DEI_max_ was successfully applied to decedents. Third, intra-observer agreement and inter-observer agreement were not tested in this study. However, a previous study [26] reported negligible observer variabilities for ROI measurements. Fourth, inter-scanner variability was not assessed in this study. Fifth, a potential benefit of using the dual-energy technique could not be assessed since the dual-energy data do not allow ECTS reconstructions. Additionally, CT numbers above the upper limit of the ECTS had to be excluded in this study.

In conclusion, this study presents a viable approach for *in situ* distinction of bullets composed of different metallic components according to their x-ray attenuation characteristics at two different energy levels. If the bullet geometry is not visually identifiable due to deformation or fragmentation, then *in situ* classification of bullets according to their metallic components can provide rapid information on the type of bullet. Since CT scanning is routinely performed on shooting victims in emergency hospitals [30, 31] and increasingly applied postmortem in forensic medicine [32–34], *in situ* distinction between copper and lead bullets is becoming increasingly feasible.

## Abbreviations

CT: Computed tomography
DEI: Dual-energy index
ECTS: Extended CT scale
HU: Hounsfield unit
ROI: Region of interest
